# Molecular Characterization and Meta-Analysis of Gut Microbial Communities Illustrate Enrichment of Prevotella and Megasphaera in Indian Subjects

**DOI:** 10.3389/fmicb.2016.00660

**Published:** 2016-05-09

**Authors:** Shrikant Bhute, Pranav Pande, Sudarshan A. Shetty, Rahul Shelar, Sachin Mane, Shreyas V. Kumbhare, Ashwini Gawali, Hemal Makhani, Mohit Navandar, Dhiraj Dhotre, Himangi Lubree, Dhiraj Agarwal, Rutuja Patil, Shantanu Ozarkar, Saroj Ghaskadbi, Chittaranjan Yajnik, Sanjay Juvekar, Govind K. Makharia, Yogesh S. Shouche

**Affiliations:** ^1^Department of Zoology, Savitribai Phule Pune UniversityPune, India; ^2^Microbial Culture Collection, National Centre for Cell Sciences, Savitribai Phule Pune University campusPune, India; ^3^Diabetes Unit, KEM Hospital Research CentrePune, India; ^4^Vadu Rural Health Program, KEM Hospital Research CentrePune, India; ^5^Department of Anthropology, Savitribai Phule Pune UniversityPune, India; ^6^Department of Gastroenterology and Human Nutrition, All India Institute of Medical SciencesNew Delhi, India

**Keywords:** Indian subjects, 16S rRNA amplicon, qPCR, *Prevotella* and *Megasphaera*

## Abstract

The gut microbiome has varied impact on the wellbeing of humans. It is influenced by different factors such as age, dietary habits, socio-economic status, geographic location, and genetic makeup of individuals. For devising microbiome-based therapies, it is crucial to identify population specific features of the gut microbiome. Indian population is one of the most ethnically, culturally, and geographically diverse, but the gut microbiome features remain largely unknown. The present study describes gut microbial communities of healthy Indian subjects and compares it with the microbiota from other populations. Based on large differences in alpha diversity indices, abundance of 11 bacterial phyla and individual specific OTUs, we report inter-individual variations in gut microbial communities of these subjects. While the gut microbiome of Indians is different from that of Americans, it shared high similarity to individuals from the Indian subcontinent i.e., Bangladeshi. Distinctive feature of Indian gut microbiota is the predominance of genus *Prevotella* and *Megasphaera*. Further, when compared with other non-human primates, it appears that Indians share more OTUs with omnivorous mammals. Our metagenomic imputation indicates higher potential for glycan biosynthesis and xenobiotic metabolism in these subjects. Our study indicates urgent need of identification of population specific microbiome biomarkers of Indian subpopulations to have more holistic view of the Indian gut microbiome and its health implications.

## Introduction

The gut microbial ecosystem is known to be governed by ecological and evolutionary forces (Ley et al., 2006) and is often controlled by secretions from the host at intestinal epithelium-microbiota interface such that beneficial microbes are maintained (Schluter and Foster, 2012). The physiological diversity of gut microbiota and its role in human health has been an inspiration for the initiation of elite projects such as Human Microbiome Project (HMP; Turnbaugh et al., 2007) and Metagenomics of the Human Intestinal Tract (MetaHIT) project (Qin et al., 2010). These projects and other related studies have generated wealth of information suggesting a link between gut microbiota and their genomic capabilities in maintenance of general wellbeing (Cho and Blaser, 2012) and also in highly specialized functions such as development of the immune system (Chung et al., 2012), neurodevelopmental disorders (Hsiao et al., 2013), and xenobiotic metabolism (Maurice et al., 2013).

Studies in past few years have highlighted discernible patterns of gut microbiota and microbiome in geographically separated populations (Mueller et al., 2006; De Filippo et al., 2010; Nam et al., 2011; Yatsunenko et al., 2012). Such studies are important in light of possible role of gut microbiota in the modulation of efficacy of oral vaccines (Valdez et al., 2014). In addition, action of pre and probiotics varies based on type of prebiotic, strain of probiotics used and possibly host gut environment (Boyle et al., 2006). Population specific microbiota studies such as American Gut, Canadian Microbiome, Brazilian Microbiome project and others are likely to yield valuable information about the gut microbiota as a target for medical interventions, may be in the form of fecal microbial transplantation to restore the healthy state (Borody et al., 2014).

Indian population is a unique conglomeration of genetically diverse groups having varied dietary habits and residing in vast geographic locations (Basu et al., 2003; Xing et al., 2010). In addition to these genetic differences, Indians have distinctive metabolic (Shukla et al., 2002) and anthropometric features (Yajnik et al., 2003; Prasad et al., 2011). Moreover, Indians are also confronted with the double burden of under- and over-nutrition primarily due to the income inequalities (Subramanian et al., 2007). In this study, we provide detailed account of prominent attributes of the Indian gut microbial composition and its functions from 34 healthy Indian subjects. We carried out 16S rRNA gene amplicon sequencing using different sequencing platforms *viz*. Ion Torrent PGM and Illumina HiSeq. We then combined the 16S rRNA amplicon data of Indian subjects together with American (Muegge et al., 2011), Korean (Nam et al., 2011), Spanish (Peris-Bondia et al., 2011), and Bangladeshi (Lin et al., 2013) to compare it with gut microbiota of these populations. In addition, considering the response of gut microbiota to different types of diets; we compared Indian gut microbiota with non-human primates including hind-gut-fermenters, fore-gut-fermenters, herbivorous, and carnivorous organisms (Ley et al., 2008).

## Materials and methods

### Study population, sample collection, and DNA extraction

We included 34 healthy Indian subjects from two urban cities: Delhi and Pune (one from Northern and one from Western part) of India and nearby rural regions of these cities. These cities are characterized by diverse groups of individuals from different parts of the country. Institutional Ethical Committee of National Centre for Cell Science approved the study and informed consent was obtained form all the participants. Although, this was not a clinical trial, we followed all good clinical practices as per Indian Council of Medical Research guidelines while recruiting the subjects and throughout the study. Fecal samples were collected from all of the subjects and stored at −80°C until DNA extraction. Total community DNA was extracted from each fecal sample using QIAmp DNA Stool Mini kit (Qiagen, Madison USA) as per manufacturer's instructions.

### 16S rRNA gene amplicon sequencing

16S rRNA amplicon sequencing of samples from Western region was performed using Ion Torrent PGM and that from Northern region using Illumina Hiseq2000 sequencing technology. For Ion Torrent PGM sequencing, samples were processed as follows: PCR was set up in 50 μl reaction using AmpliTaq Gold PCR Master Mix (Life Technologies, USA) and with 16S rRNA V3 region specific bacterial universal primers: forward primer 341F (5′-CCTACGGGAGGCAGCAG-3′) and reverse primer 518R (5′-ATTACCGCGGCTGCTGG-3′; Bartram et al., 2011). Following conditions were used for PCR: initial denaturation at 95°C for 4 min, followed by 20 cycles of 95°C for 1 min, 56°C for 30 s, and 72°C for 30 s with a final extension at 72°C for 10 min. PCR products were purified using Agencourt AMPure XP DNA purification Bead (Beckman Coulter, USA), end repaired and ligated with specific barcode adaptor as explained in *Ion Xpress*™ *Plus gDNA Fragment Library Preparation* user guide. Fragment size distribution and molar concentrations of amplicon were assessed on a Bioanalyzer 2100 (Agilent Technologies, USA) using High Sensitivity DNA Analysis Kit as per manufacturer's instructions. Emulsion PCR was carried out on diluted and pooled amplicon (10 samples in each pool) using the Ion OneTouch™ 200 Template Kit v2 DL (Life Technologies). Sequencing of the amplicon libraries was carried out on 316 chips using the Ion Torrent PGM system and Ion Sequencing 200 kit (Life Technologies). For Illumina sequencing, samples were processed as follows: A PCR reaction of 50 μl was set up using AmpliTaq Gold high fidelity polymerase (Life Technologies, USA) and PCR conditions were as follows: initial denaturation at 95°C for 10 min; followed by 30 cycles of 95°C for 30 s; 56°C for 30 s; and 72°C for 30 s. The final extension was set at 72°C for 7 min. The PCR products were purified using gel elution and the eluted products were used for library preparation. The libraries were quantified on Bioanalyzer using the DNA high sensitivity LabChip kit (Agilent Technologies, USA) and sequenced using Illumina HiSeq2000 (2x150 PE).

### Sequence processing and bioinformatics analysis

All PGM and Illumina HiSeq reads were pre-processed using Mothur pipeline (Schloss et al., 2009) with following conditions: minimum 150 bp to maximum 200 bp, maximum homopolymer–5, maximum ambiguity–0, and average quality score–20. This way we derived total of ~17 million high quality amplicon reads from 34 samples, which we pooled into single FASTA file for further analysis in QIIME: Quantitative Insights Into Microbial Ecology (Caporaso et al., 2010). Closed reference based OTU picking approach was used to cluster reads into Operational Taxonomic Units (OTUs) at 97% sequence similarity using UCLUST algorithm (Edgar, 2010) and a representative sequence from each OTU was selected for downstream analysis. All OTUs were assigned to the lowest possible taxonomic rank by utilizing RDP Classifier 2.2 (Wang et al., 2007) and Greengenes database 13.8 with a confidence score of at least 80%. Estimations of Core OTUs were done as described previously (Huse et al., 2012). Various estimates of alpha diversity such as Chao1, PD whole tree, Simpson, and Shannon were applied on rarefied sequence count (1181 sequence per sample) and UniFrac was used as beta diversity measures to understand the microbial communities in Indian individuals. UniFrac analysis is known to be affected by sequencing depth and evenness, therefore, we performed jackknifing in which samples are subjected to even subsampling for n replicates and UniFrac distance matrix is calculated for each replicate (Lozupone and Knight, 2005). This way we generated 1000 replicates of PCoA coordinates and Procrustes analysis was applied to each PCoA replicate to plot average position of individuals on PCoA plot. The interquartile range of the distribution of points among the replicates was represented as an eclipse around the point (Lozupone et al., 2011).

### qPCR based quantification of dominant OTUs

The abundance of intestinal bacterial groups belonging to genus *Prevotella, Faecalibacterium*, and *Megasphaera* were measured by absolute quantification of 16S rRNA gene copy number by using primers listed in Supplementary Table 1. Template concentration for each sample was initially adjusted to 50 ng/μl. qPCR amplification and detection were performed in 10 μl reaction (consisting of 5 μl Power SYBR Green PCR Master Mix, 0.1 μM of each specific primer and 1 μl template) in triplicate using 7300 Real time PCR system (Applied Biosystems Inc., USA). Following conditions were used for qPCR assays: one cycle of 95°C 10 min followed by 40 cycles of 95°C for 15 s and 60°C for 1 min. Group specific standard curves were generated from 10-fold serial dilutions of a known concentration of PCR products for each group. Average values of the triplicate were used for enumerations of 16S rRNA gene copy numbers for each group using standard curves generated (Marathe et al., 2012). Percent abundance of each genus was obtained by calculating ratio of copy number of that genus to that of total bacteria. Throughout the qPCR experiments efficiency was maintained above 90% with a correlation coefficient >0.99.

### Imputation of metagenome using PICRUSt

The metagenome imputation was done using method as described earlier (Langille et al., 2013). Briefly, closed reference based OTU picking approach was utilized to bin the amplicon sequences using latest Greengenes database 13.5 at 97% sequence similarity cut-off. The normalization for 16S rRNA gene copy number was carried out before prediction of the metagenome. This OTU table was used for predicting metagenome at three different KEGG levels (L1 to L3). Metagenomic differences between Indians-Americans as well as Indian-non-human primates were analyzed using linear discriminant analysis (LDA) effect size (LEfSe; Segata et al., 2011). PICRUSt and LEfSe analysis were performed with available parameters at http://huttenhower.sph.harvard.edu/galaxy/.

### Publically available data used

We did a PubMed search restricted only to publically available 16S rRNA amplicon data. Upon further narrowing down our search, we obtained raw sequence data of Korean subjects (DDJB project ID 60507; Nam et al., 2011), Bangladeshi subjects (SRA-SRA057705; Lin et al., 2013), data of 18 American individuals and 33 non-human primates (MG-RAST qiime625 and qiime626; Muegge et al., 2011) and data of Spanish individuals (SRA-SRP005393; Peris-Bondia et al., 2011). The list of primers, variable region of 16S rRNA gene and sequencing technology for each of the study is listed in Supplementary Table 2. Any previously reported sequence data for Indian population was not available. To avoid biases introduced due to respective studies describing microbiota of these populations and inter-individual variations, sequence data of all individuals from a study was merged and considered as a representative microbiota of that country. The raw data from all these samples was processes along with the Indian sequence data (Ion Torrent and Illumina amplicons) in the same way as explained earlier.

### Additional statistical tests

We applied Good's coverage to have a sense of understanding that the sequencing we have performed was enough to cover microbial diversity in the samples studied (Good, 1953). We also applied Welch's *t*-test with Benjamini-Hochberg FDR correction to examine the significantly differing bacterial families between Indians and Americans and Kruskal-Wallis test (a non-parametric measure of variance) to examine the population specific OTUs. Similar comparisons were made to evaluate the differential OTUs among non-human primates and Indians. Random Forest, a supervised machine-learning approach was applied to our data sets to identify taxa that were indicators for community differences in Indians-Americans as well as Indian-non-human primates (Knights et al., 2011; Yatsunenko et al., 2012). An OTU was given importance scores by estimating amount of error introduced if that OTU is removed from the set of indicator taxa.

## Results

### Key features of Indian gut microbiota

We obtained over 17 million good quality reads which were clustered into 3782 OTUs from the 34 healthy Indian individuals, for further analysis the sequences were normalized to 1181 per sample (Supplementary Table 3). We first employed Good's coverage in order to estimate that enough sequencing has been performed to address the gut microbial diversity; with mean Good's coverage of 94% ±0.03, we were convinced of capturing dominant OTUs in all study subjects and to comment on gut microbial features of them.

**Figure 1:**
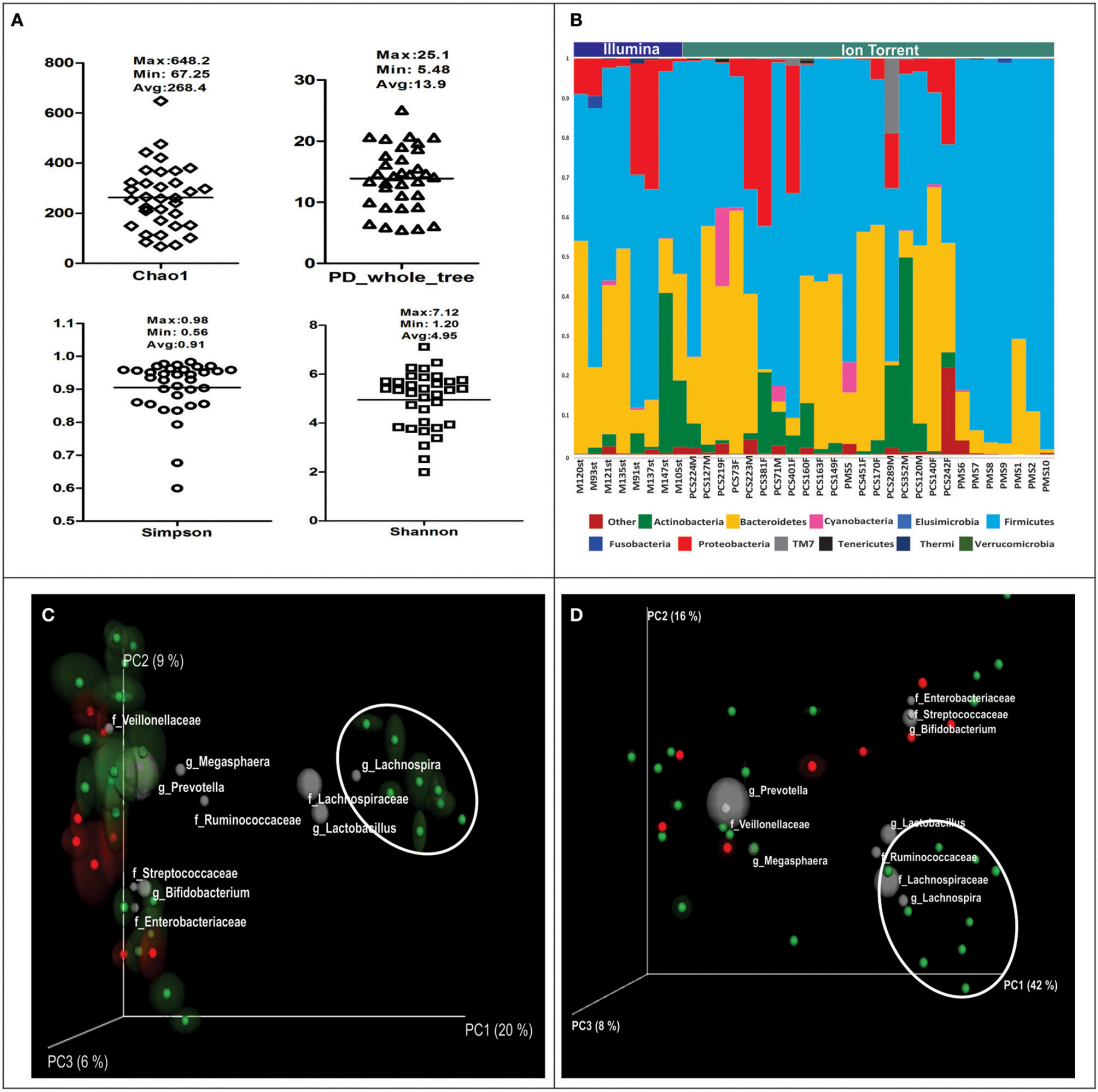
**(A)** Variation in alpha diversity indices in Indian Subjects. **(B)** Abundance of dominant bacterial phyla in Indian subjects. Subjects are separated and shown according to sequencing platform used. Samples with prefix PMS are from rural region and rest are from urban region. **(C)** Unweighted and **(D)** weighted UniFrac PCoA bi-plots; the gray colored sphere represent a taxonomic group that influence clustering of samples in particular area of the PCoA plot and its size demonstrate abundance of that taxonomic group (Rural samples are encircled). Colors indicate the sequencing technology used. Red: Illumina, Green: Ion Torrent PGM.

We used alpha diversity indices to understand community composition of gut microbiota, some of which were based on species richness and species abundance and some on phylogenetic distance between them. Alpha diversity indices such as Chao1, Shannon, Simpson, and PD_Whole tree revealed that there were large differences in the community composition in study subjects under consideration (Figure 1A). Upon comparison of alpha diversity indices between rural and urban population, it was observed to be higher in urban subjects, however, no significant differences were noted for alpha diversity indices with respect to sequencing technology used. Overall, we could detect 201 bacterial genera belonging to 11 bacterial phyla in Indian subjects (Figure 1B). Upon closer examination of the OTU table we were able to detect 50 OTUs that were present across the samples, such OTUs are commonly termed as core OTUs (Table 1). Presence of just 50 core OTUs suggest that the gut microbiome of Indians is very diverse. This was further confirmed by performing beta diversity analysis using unweighted (sensitive to presence of unique OTUs) and weighted (sensitive to the abundance) UniFrac distance matrices. In each case, jackknifed PCoA biplots were produced to illustrate the compositional variation in gut microbiota between the samples; position of each sample is the average of jackknifed replicate shown with ellipses representing the IQR in each axis. Presence of large ellipses around each sample sphere in unweighted PCoA plot (Figure 1C) is indicative of variations on beta diversity measures due to random subsampling and thus the presence of unique OTUs particular to each individual. Interestingly, we also noted that the samples that were happened to be collected from rural areas (eight samples on the right side of Figure 1C) clustered separately from the urban samples on unweighted PCoA plot indicating the contribution of lifestyle associated factors on sample segregation. However, on weighted PCoA plot (Figure 1D), all samples found scattered indicating the abundance of taxa influencing the segregation of samples on weighted PCoA plot was not different among the samples. Further, from the taxa contributing sample segregation of PCoA plots and from core OTUs, it was noticed that the gut microbiota of Indians is highly enriched with the OTUs belonging to bacterial genera *Prevotella* and *Megasphaera* and bacterial families such as Lachnospiraceae, Ruminococcaceae, and Veillonellaceae.

**Table 1:** **Showing the core OTUs found in Indian subjects**.

**OTU ID**	**OTU Taxonomy**
825808	Bacteria|Actinobacteria|Actinobacteria|Bifidobacteriales|Bifidobacteriaceae|Bifidobacterium
3177392	Bacteria|Actinobacteria|Actinobacteria|Bifidobacteriales|Bifidobacteriaceae|Bifidobacterium|longum
4347159	Bacteria|Actinobacteria|Actinobacteria|Bifidobacteriales|Bifidobacteriaceae|Bifidobacterium
178860	Bacteria|Bacteroidetes|Bacteroidia|Bacteroidales|Prevotellaceae|Prevotella|copri
292041	Bacteria|Bacteroidetes|Bacteroidia|Bacteroidales|Prevotellaceae|Prevotella|copri
196729	Bacteria|Bacteroidetes|Bacteroidia|Bacteroidales|Prevotellaceae|Prevotella|copri
189521	Bacteria|Bacteroidetes|Bacteroidia|Bacteroidales|Prevotellaceae|Prevotella|copri
4410166	Bacteria|Bacteroidetes|Bacteroidia|Bacteroidales|Prevotellaceae|Prevotella|copri
4295618	Bacteria|Bacteroidetes|Bacteroidia|Bacteroidales|Prevotellaceae|Prevotella|copri
4436552	Bacteria|Bacteroidetes|Bacteroidia|Bacteroidales|Prevotellaceae|Prevotella|copri
189083	Bacteria|Bacteroidetes|Bacteroidia|Bacteroidales|Prevotellaceae|Prevotella|copri
4406684	Bacteria|Bacteroidetes|Bacteroidia|Bacteroidales|Prevotellaceae|Prevotella|copri
4410166	Bacteria|Bacteroidetes|Bacteroidia|Bacteroidales|Prevotellaceae|Prevotella|copri
4463108	Bacteria|Firmicutes|Bacilli|Lactobacillales|Lactobacillaceae|Lactobacillus|ruminis
4463108	Bacteria|Firmicutes|Bacilli|Lactobacillales|Lactobacillaceae|Lactobacillus|ruminis
4392549	Bacteria|Firmicutes|Bacilli|Lactobacillales|Streptococcaceae
288417	Bacteria|Firmicutes|Bacilli|Lactobacillales|Streptococcaceae|Streptococcus|luteciae
2814830	Bacteria|Firmicutes|Clostridia|Clostridiales
334340	Bacteria|Firmicutes|Clostridia|Clostridiales|Lachnospiraceae
210092	Bacteria|Firmicutes|Clostridia|Clostridiales|Lachnospiraceae
551141	Bacteria|Firmicutes|Clostridia|Clostridiales|Lachnospiraceae
327383	Bacteria|Firmicutes|Clostridia|Clostridiales|Lachnospiraceae
309564	Bacteria|Firmicutes|Clostridia|Clostridiales|Lachnospiraceae
213487	Bacteria|Firmicutes|Clostridia|Clostridiales|Lachnospiraceae
188918	Bacteria|Firmicutes|Clostridia|Clostridiales|Lachnospiraceae
4087649	Bacteria|Firmicutes|Clostridia|Clostridiales|Lachnospiraceae
194870	Bacteria|Firmicutes|Clostridia|Clostridiales|Lachnospiraceae
334340	Bacteria|Firmicutes|Clostridia|Clostridiales|Lachnospiraceae
188918	Bacteria|Firmicutes|Clostridia|Clostridiales|Lachnospiraceae
335789	Bacteria|Firmicutes|Clostridia|Clostridiales|Lachnospiraceae|Blautia
362568	Bacteria|Firmicutes|Clostridia|Clostridiales|Lachnospiraceae|Blautia
335789	Bacteria|Firmicutes|Clostridia|Clostridiales|Lachnospiraceae|Blautia
296010	Bacteria|Firmicutes|Clostridia|Clostridiales|Lachnospiraceae|Dorea
330458	Bacteria|Firmicutes|Clostridia|Clostridiales|Lachnospiraceae|Roseburia
330458	Bacteria|Firmicutes|Clostridia|Clostridiales|Lachnospiraceae|Roseburia
4483037	Bacteria|Firmicutes|Clostridia|Clostridiales|Ruminococcaceae
368117	Bacteria|Firmicutes|Clostridia|Clostridiales|Ruminococcaceae
315978	Bacteria|Firmicutes|Clostridia|Clostridiales|Ruminococcaceae
4483037	Bacteria|Firmicutes|Clostridia|Clostridiales|Ruminococcaceae
315978	Bacteria|Firmicutes|Clostridia|Clostridiales|Ruminococcaceae
361966	Bacteria|Firmicutes|Clostridia|Clostridiales|Ruminococcaceae|Faecalibacterium
4341534	Bacteria|Firmicutes|Clostridia|Clostridiales|Ruminococcaceae|Faecalibacterium
2386814	Bacteria|Firmicutes|Clostridia|Clostridiales|Ruminococcaceae|Faecalibacterium
199430	Bacteria|Firmicutes|Clostridia|Clostridiales|Ruminococcaceae|Faecalibacterium
361966	Bacteria|Firmicutes|Clostridia|Clostridiales|Ruminococcaceae|Faecalibacterium
199430	Bacteria|Firmicutes|Clostridia|Clostridiales|Ruminococcaceae|Faecalibacterium
4308811	Bacteria|Firmicutes|Clostridia|Coriobacteriales|Coriobacteriaceae
4481613	Bacteria|Firmicutes|Clostridia|Coriobacteriales|Coriobacteriaceae|Collinsella|aerofaciens
4481613	Bacteria|Firmicutes|Clostridia|Coriobacteriales|Coriobacteriaceae|Collinsella|aerofaciens
2072645	Bacteria|Proteobacteria|Gammaproteobacteria|Enterobacteriales|Enterobacteriaceae|Escherichia

**Figure 2:**
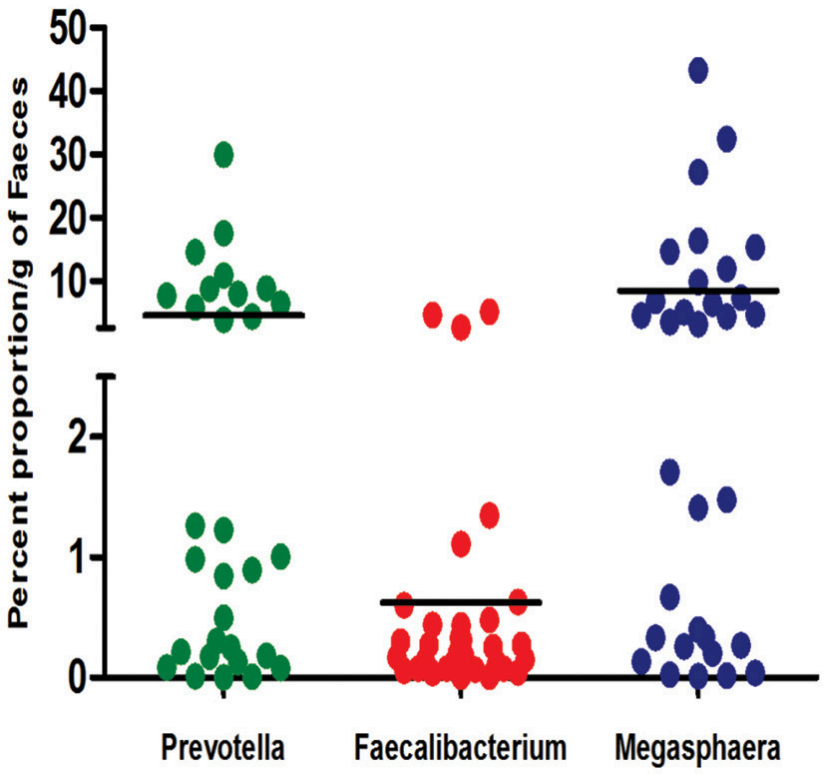
**16S rRNA gene copy number of bacterial genera of most and least abundant bacterial OTUs in Indian subjects**. The results are expressed as percent abundance of Prevotella, Faecalibacterium and Megasphaera to that of the total bacteria for each of the sample.

To confirm that Indian gut microbiota is enriched with *Prevotella* and *Megasphaera* OTUs, we carried out qPCR assays for absolute quantification of 16S rRNA copy number of these genera in the study subjects. Mean count of *Prevotella* and *Megasphaera* was found to be 4.45% and 8.45%, respectively of total bacterial count. On the contrary, *Faecalibacterium* mean count was as low as 0.63% of total bacterial count (Figure 2). Interestingly, based on absolute count of *Prevotella* and *Megasphaera* Indian subjects were demarcated into two groups, one with moderate and other with high copy number of these genera. These results confirmed the 16S rRNA gene amplicon analysis and signify the dominance of *Prevotella* and *Megasphaera* in Indians.

### Quantitative differences between gut microbiota of Indians and Americans

**Figure 3:**
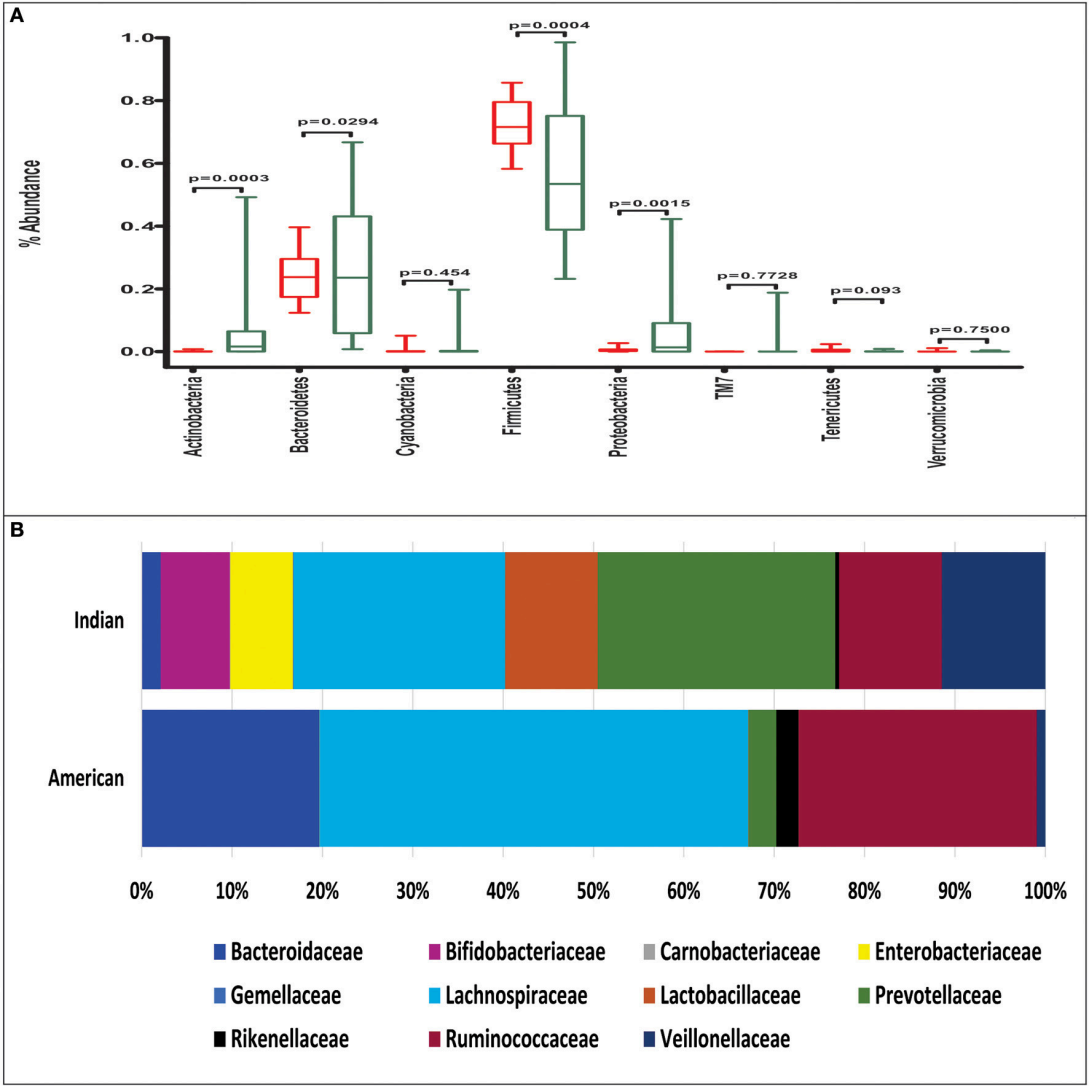
**(A)** Phylum level abundance of gut microbiota in Indians (green box) and Americans (red box), the boxes represent interquartile range (IQR), and the line between boxes indicate median value. **(B)** Abundance of significantly different bacterial families in Indians and Americans (Welch's *t*-test *P*-value below 0.05).

**Figure 4:**
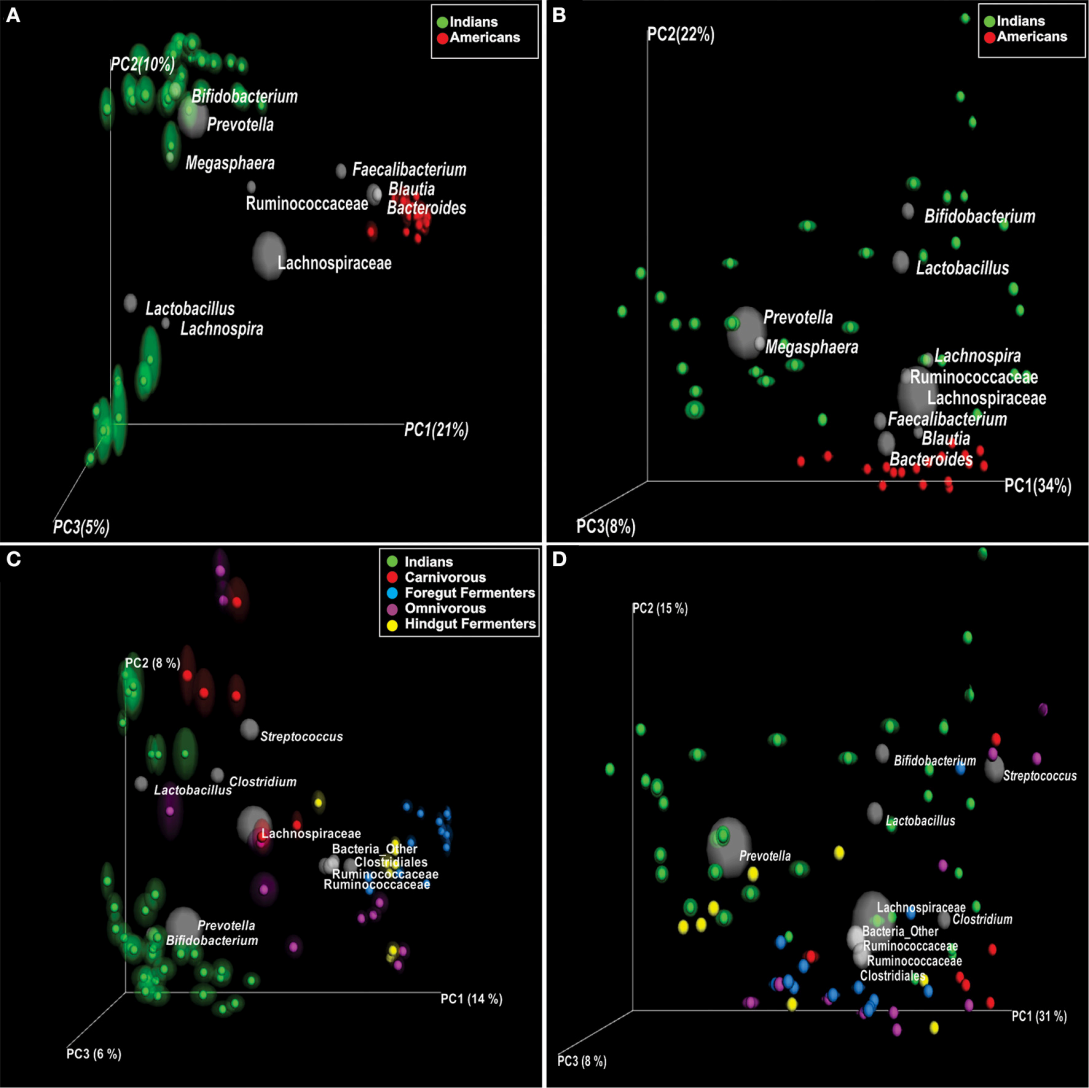
**Principal coordinate analysis (PCoA) of UniFrac distance matrices; samples are colored according to the country (A,B) and according to the diet for comparison with non-human primates (C,D)**. **(A)** unweighted and **(B)** weighted UniFrac PCoA bi-plots; showing clustering of Indians and Americans, the gray colored sphere represent a taxonomic group that influence clustering of these samples in particular area of PCoA plot and its size demonstrate abundance of that taxonomic group. **(C)** Unweighted and **(D)** weighted UniFrac PCoA bi-plots; showing clustering of Indians and non-human primates.

The mean abundance of bacterial phyla and families between Indians and Americans was compared using *t*-test. Significant differences were observed in four dominant phyla in these populations: Actinobacteria (*P* = 0.0003), Bacteroidetes (*P* = 0.029), and Proteobacteria (*P* = 0.0015) being significantly more abundant in Indians and Firmicutes (*P* = 0.0004) in Americans (Figure 3A). At family level, 11 families were observed to be significantly different in the two populations (Figure 3B and Supplementary Table 4). Prevotellaceae, Lactobacillaceae, Veillonellaceae, Bifidobacteriaceae, Enterobacteriaceae, Streptococcaceae, Peptostreptococcaceae, Leuconostocaceae, Micrococcaceae, Carnobacteriaceae, and Gemellaceae were more dominant in Indians (*P* < 0.05) whereas Lachnospiraceae, Ruminococcaceae, Bacteroidaceae, Coprobacillaceae, Porphyromonadaceae, Rikenellaceae, Erysipelotrichaceae, Desulphovibrionaceae, and Christensenellaceae were more dominant in Americans (*P* < 0.05). Kruskal-Wallis test revealed total of 127 OTUs differed significantly (*P* < 0.01) between the two populations (Supplementary Table 5) of which 50 were unique to Indians and 475 to Americans. Principal coordinate analysis (PCoA) based on weighted and unweighted UniFrac distance matrices demonstrated that the Americans clustered along coordinate 1 (Figures 4A,B) as against the Indians who were found dispersed along the coordinate 2. For Random Forest analysis, we considered an OTU to be highly predictive if its importance score was at least 0.001, this revealed 76 highly predictive OTUs between the two populations (Supplementary Table 6). Among these 76 highly predictive OTUs, 6 were overrepresented in Indians while rest were overrepresented in Americans. The OTUs overrepresented in Indians belonged to genus *Prevotella, Lactobacillus, Lachnospira* and *Roseburia*. Our results highlight profound differences at various taxonomic levels in gut microbial community structure of the two populations.

**Figure 5:**
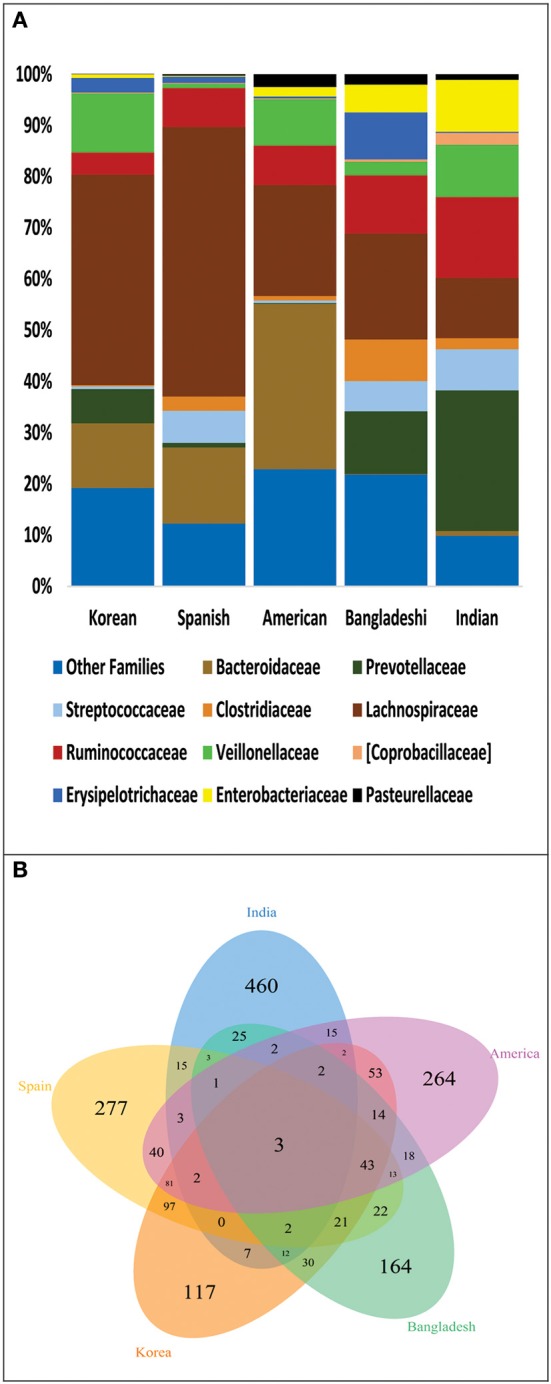
**(A)** Family level distribution of bacterial taxa among: Indian, Bangladeshi, American, Korean, and Spanish population. **(B)** Venn diagram demonstrating overlap of OTUs at 97% sequence similarity cut off among these populations.

We further analyzed the differences in gut microbiota of Indian, Bangladeshi, American, Korean and Spanish populations in terms of unique and shared bacterial families plus the OTUs among these populations. For this, we normalized the sequence data to 4389 sequences per sample which contributed to 1807 OTUs. At bacterial family level, Indians shared more families with Bangladeshis; while fewer with Americans, Koreans and Spanish (Figure 5A). With 460 unique OTUs (Supplementary Table 7), Indians shared maximum of 25 OTUs with Bangladeshi, 15 with Americans and Spanish while 7 with Koreans (Figure 5B). Most of the shared OTUs between Indians and Bangladeshis belonged to families Lachnospiraceae, Ruminococcaceae and Enterobacteriaceae, and genus *Prevotella* (Supplementary Table 8). Interestingly, only 3 OTUs were common in all populations, which were contributed by Streptococcaceae and Enterobacteriaceae families.

### Indians share microbiota with omnivorous mammals

**Figure 6:**
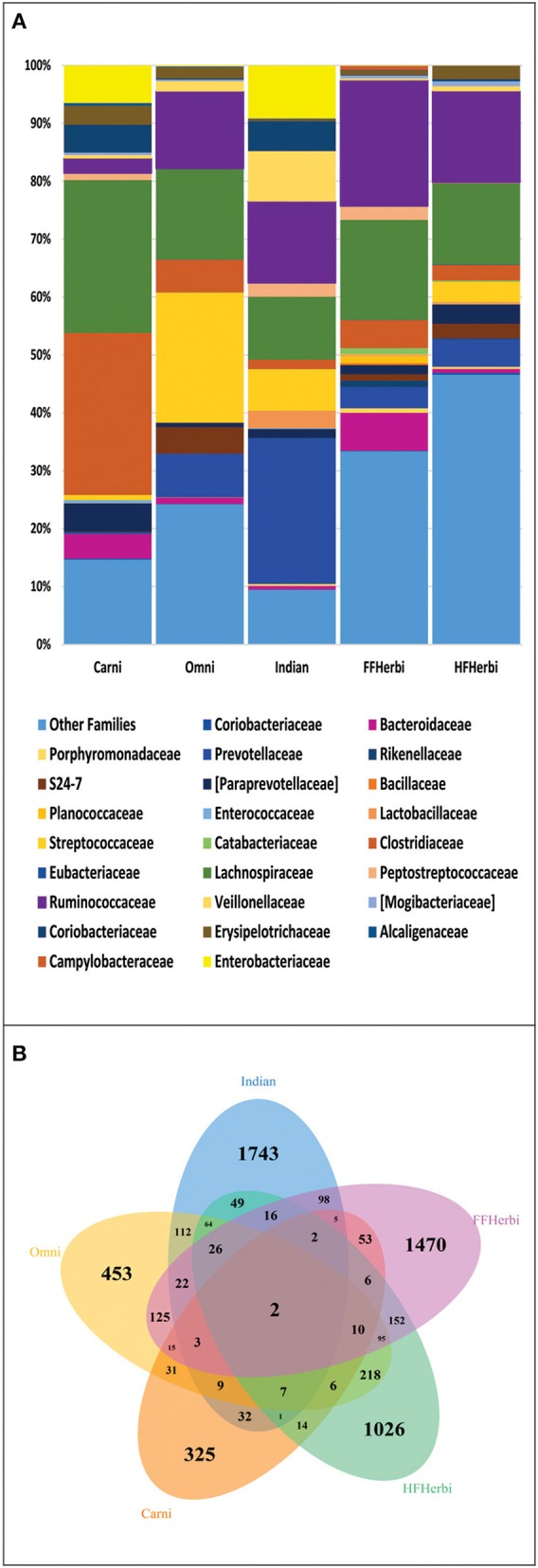
**(A)** Family level distribution of bacterial taxa among: Indians and non-human primates. **(B)** Venn diagram demonstrating overlap of OTUs at 97% sequence similarity cut off among Indians and non-human primates.

For the comparison of gut microbiota of Indians with non-human primates, we normalized the sequences to 1181 sequences per sample, which constituted 6189 OTUs. We observed that Indians share maximum 68 of 236 bacterial families (Figure 6A) and 112 OTUs with omnivorous mammals (Supplementary Table 9) while minimum of 32 OTUs with carnivorous mammals. Interestingly, only 2 OTUs were common in all non-human primates and Indians (Figure 6B). Further, principal coordinate analysis (PCoA) based on unweighted and weighted UniFrac distance matrices showed scattered distribution of omnivorous samples (Figures 4C,D). On the contrary, herbivorous (hind gut and fore gut fermenters), and carnivorous clustered separately (Figure 4C). Random Forest analysis of Indians and non-human primates revealed 652 highly discriminants OTUs. Of the 341 OTUs, 122 and 174 OTUs were overrepresented in Indians and omnivorous mammals, respectively (Supplementary Table 10).

### Imputed metagenome

**Figure 7:**
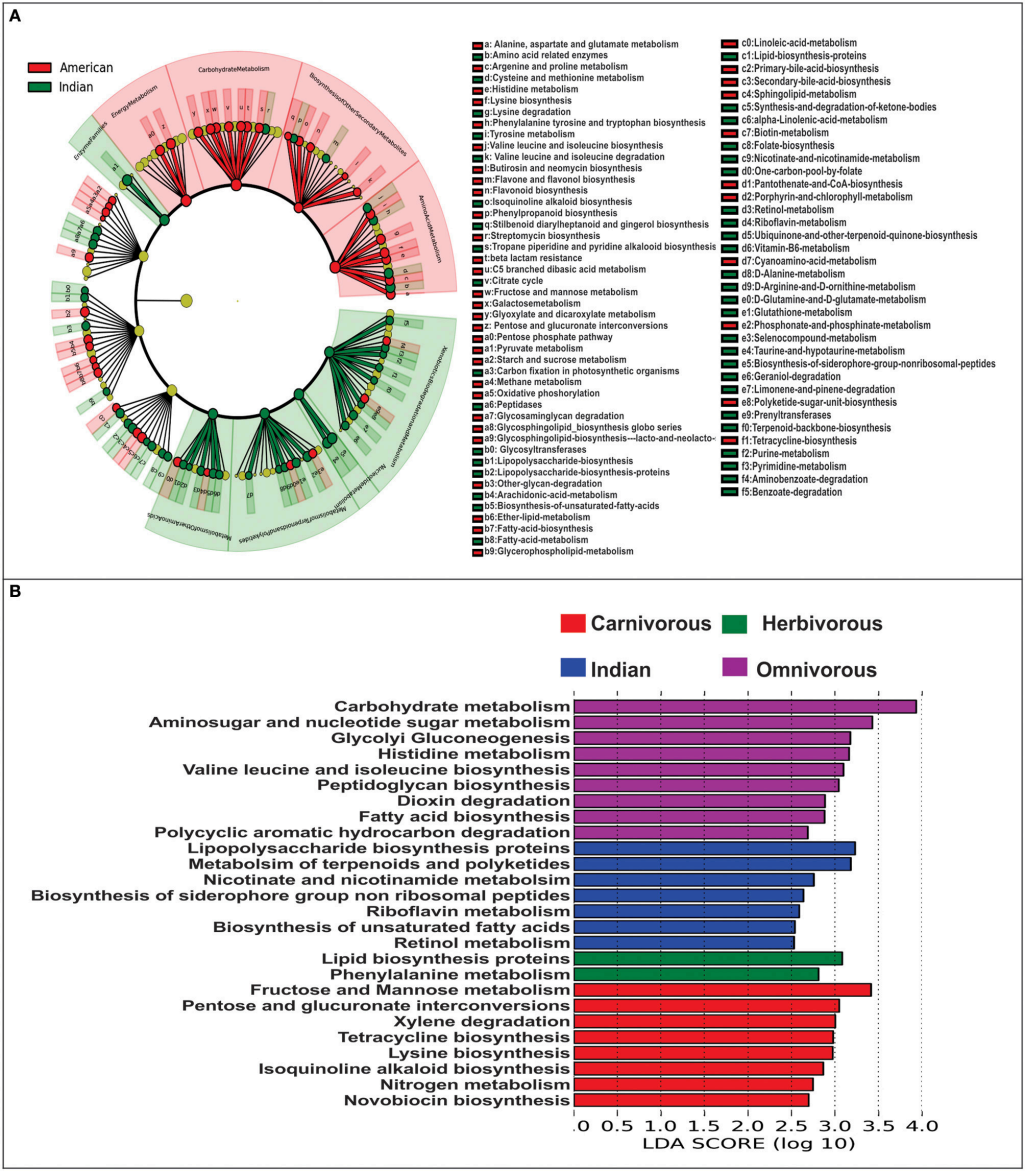
**Metagenomic imputation**. **(A)** Cladogram from LEfSe analysis representing differentially abundant KOs between Indians and Americans. **(B)** Visualization of unique metabolic functions among the Indians and other non-human primates.

For comparing functional potential of the microbial communities in Indians and Americans, we used PICRUSt (Phylogenetic Investigation of Communities by Reconstruction of Unobserved States). PICRUSt uses extended ancestral-state reconstruction algorithm to estimate which gene families are present and then combines gene families to give complete metagenome of the samples. From the data of functional capabilities, we focused primarily on those, which are associated with the microbial metabolism. We noticed significant differences in all major metabolic functions in gut microbiome of Indians and Americans. Broadly, gene families associated with xenobiotic biodegradation, nucleotide metabolism, enzyme families, metabolism of terpenoids and polyketides, glycan biosynthesis, and metabolism were overrepresented in Indians, whereas, metabolic functions associated with energy metabolism, carbohydrate metabolism, amino acid metabolism, and biosynthesis of other secondary metabolites were overrepresented in Americans (Figure 7A). Further, the metagenomic comparison between Indians and non-human primates revealed that gene families linked to energy harvesting potential such as carbohydrate metabolism, glycolysis-gluconeogenesis, and fatty acid biosynthesis were enriched in omnivorous mammals (Figure 7B).

## Discussion

Studies concerning population specific microbiota have revealed peculiar patterns in distribution of specific microbial communities in their gut. Surprisingly, till date, no efforts have been made to understand specific features of the microbiota of healthy Indian subjects. Based on 16S rRNA data from 34 individuals and 3782 OTUs, in this work, we first systematically describe gut microbiota features in Indian subjects. We suggest vast inter-individual variation in gut microbial communities in these subjects, characterized by dominance of *Prevotella* and *Megasphaera*. We further demonstrate the graded difference in microbial communities of these subjects from neighboring country (Bangladeshi) to distant population (Americans) as well as show that they indeed share most of the microbiota with omnivorous animals.

Our observation of compositional and phylogenetic variation within Indian gut microbiota as revealed by alpha diversity indices, could be a result of different variables like biogeographic separations of individuals (like rural-urban setting) and associated life-style factors. Further, we noted large variation in alpha diversity indices in urban individuals. Thus, to check whether there are unique taxa responsible for this, we performed UniFrac based beta diversity analysis. Indeed, the separation between rural and urban subjects observed on unweighted UniFrac PCoA which is influenced by less abundant unique OTUs was lost on weighted UniFrac PCoA because of abundance of dominant OTUs. The distinct separation was also not evident at phyla level abundance. On PCoA bi-plots, we further showed the contribution of dominant taxonomic groups influencing the segregation of samples. Thus, our results are robust and proves the presence of individual specific OTUs; at the same time it confirms that Indian subjects could not be separated into two or more groups based on presence and abundance of dominant taxonomic groups.

Knowing the fact that gut microbiota is influenced by diet and geography, we extended our analysis and compared gut microbiota of Indian subjects with American gut microbiota. Based on composition of microbiota, Americans were closely clustered while Indians were found dispersed on PCoA-bi-plots. This distinctive clustering could be partly because of genetic make-up and largely due the calorie restricted diet that these subjects were following. Interestingly, in an another study Americans from metropolitan areas which were not on any specific diet, segregated distantly from those of Malawians and Amerindians and were clustered closely (Yatsunenko et al., 2012). This provides the clue that though the cohort was calorie restricted, gut microbiota of Americans is indeed different from gut microbiota of other communities. Thus, diet can be one of many factors which influence the gut microbial communities and other factors such as genetic make-up and other current practices could also have a major influence on gut microbial composition. On broader scale, Indian population which originated from first wave of modern humans Out-of-Africa following the coastal route; and American population, which is effectively descendants of post-Columbian European migrants (Lazaridis et al., 2014), are genetically different hosts with varied dispersal histories (Macaulay et al., 2005; Mellars et al., 2013). The lack of cohesive Indian population cluster may be due to the heterogeneous representation of Indian samples from different endogamous groups experiencing diverse dietary patterns, prescriptions-proscriptions for food and food taboos that vary culturally.

Upon analysing the differentiating bacterial lineages and contributors in PCoA-biplots, we discovered that the OTUs belonging to genus *Prevotella, Lactobacillus, Bifidobacterium*, and *Megasphaera* were discriminately abundant in Indians. Members of genus *Prevotella* are known for their ability to degrade complex plant polysaccharides (De Filippo et al., 2010), thus its high abundance in Indian gut microbiota could be a result of the nature of Indian diet, which is primarily rich in plant derived preparations (Vecchio et al., 2014). Predominance of members of *Lactobacillus* and *Bifidobacterium* could be explained by the fact that fermented foods are another major components in Indian diet; these fermented foods are good source of lactic acid bacteria (Satish Kumar et al., 2013). Members of genus *Megasphaera*, a normal inhabitant of ruminant gut, have been isolated by us from gut microbiota of Indians (Shetty et al., 2013). The genome analysis and physiological characterization of these *Megasphaera* isolates highlighted their ability to produce short chain fatty acids viz. propionate, acetate, and butyrate and vitamins like of cyanocobalamin. One of the interesting observations of our study is the demarcation of Indian individuals into two groups (moderate and high copy number of *Prevotella* and *Megasphaera*). Recently, bimodal bacteria (with low and high abundance groups) in more than 1000 western individuals were reported and were predicted to be key bacterial groups associated with host health (Lahti et al., 2014). Considering the metabolic features of Prevotella and Megasphaera explained earlier and effect of different environmental factors on microbiota, they can be represented as tipping elements in Indian gut microbiota and are possibly linked with general well-being of these subjects as all the participants were healthy. However, further analysis would be needed to confirm the bimodal nature of these groups. Further we obtained the evidence for variations in gut microbiota of Indians by comparing it with gut microbiota of Spanish, Korean, Bangladeshi and American population, which are unique with respect to their dietary patterns and biogeographic locations. Indians shared maximum taxonomic groups with next-door neighbor Bangladeshi, which became progressively less with American, Spanish, and Koreans. High similarity shared between gut microbiota of Indian and Bangladeshi population is a reflection of shared ethnicity and other life-style factors between these populations. Interestingly, Indians shared least OTUs with Korean, which in turn shared maximum OTUs with Americans is in accordance with observations of previous study (Nam et al., 2011). The most intriguing finding of this analysis however, was the presence of only three common OTUs amongst all the populations, strengthening the fact that gut microbiome of geographically separated population is indeed unique and very few OTUs may contributes to core microbiome of the global population (Huse et al., 2012).

In the meta-analysis of microbial studies often comparisons are made between the data generated using different experimental protocols, hence a critical question is whether the principal conclusions derived are because of the technical differences or they are indeed biologically meaningful? Taking into account the effect of different experimental protocols including method of DNA extraction, use of specific primers and sequencing technologies, it cannot be denied that these factors could introduce some bias in the observed results (Lozupone et al., 2013). However, by the use of more stringent approach during bioinformatics analysis of amplicon (as presented in the current manuscript), it is possible to reduce such biases. The results presented in Figure 1C indicate that the segregation of samples is not due to the sequencing technologies used, but are indeed due to the large compositional differences in microbiota. Thus, such comparisons are required to identify the influence of these factors on the observed results and will bring into light the ways of optimizing the analysis protocol in order to minimize the effect of such confounding factors.

One of the major life-style factors, which characterize a population, is its dietary habits. There are abundant evidences in the literature suggesting effect of diet on microbiota (David et al., 2014; Xu and Knight, 2014). We therefore hypothesized, that gut microbiota of Indians who typically display mixed vegetarian and non-vegetarian dietary habits may be alike omnivorous mammals. The observation of the present study regarding similarities of gut microbiome of Indians and omnivorous mammals are in congruent to previous study findings (Ley et al., 2008; Muegge et al., 2011). In a study, Ley et al. showed that indigenous gut microbial communities co-diversify with their hosts and the microbial diversity increases from carnivory to omnivory to herbivory. Moreover, presence of only two common OTUs amongst all the types of dietary patterns, hint toward subtle differences and rapid trade-offs in gut microbial communities shaped by evolutionary forces in response to animal and plant diets.

Metagenomic studies of gut microbiome suggests that microbes residing in the gut have enormous genetic potential to code for functions essential for them to thrive in the gut environment and maintain homeostasis of gut ecosystem (Qin et al., 2010). To the best of our knowledge, report on experimentally derived human gut metagenomic data from adult Indian individuals is unavailable. In this context, our metagenomic imputations become minimum essential to have first glimpse at the functional capabilities of Indian individuals. Our metagenomic imputations using PICRUSt followed by LEfSe analysis reveals vast diversity in metabolic functions in these subjects. Although, the findings of differences in metabolic capabilities among the Indians-Americans and Indians-Non-human primates are based on imputed metagenome and has some limitations as explained earlier (Langille et al., 2013), we were able to capture broader functional features in gut microbiota and correlate it with the taxonomic features. Higher abundance of Bacteroidetes are generally attributed to ability to degrade xenobiotics like antibiotics (Maurice et al., 2013) and metabolism of complex glycans (Martens et al., 2009) Whereas, the Firmicutes are related to increased energy harvest through excessive carbohydrate metabolism and production of SCFAs (Turnbaugh et al., 2006). High Bacteroidetes and low Firmicutes found in Indian subjects and their correlation with metabolic abilities, indeed suggests that their gut microbiota not only differ at taxonomic level but also at the functional levels from that of Americans.

## Conclusion

Our study raises the exciting possibility that the difference in microbiota may contribute to differences in health and disease characteristics of Indian population that could be different compared to the observations in the western population. Findings of the present study will serve as a basis for large-cohort studies in near future on Indian Gut Microbiome to address the questions such as if there are specific bacterial taxa or microbial functions which can be treated as a potential target for medical intervention studies.

## Author contributions

YS and SJ conceptualized and designed the study whereas, YS also coordinated it. SB, SM, RS performed Ion torrent PGM sequencing. SS performed Illumina Hiseq sequencing. SK, AG, HM downloaded all relevant 16S rRNA sequence data. SB, PP carried out the detailed bioinformatics analysis. MN wrote the specific PERL script for bioinformatics analysis. DD coordinated the bioinformatics analysis. SO provided the anthropological insight on Indian context. SB, SG, HL, CY, DA, RP, SJ, and GM were involved in sample collection. SB and PP wrote the manuscript and all authors edited and approved the manuscript.

## Availability of sequence data

Ion Torrent PGM runs were deposited to NCBI SRA under the accession numbers SRP041693, SRP055407 and Illumina raw reads to DDBJ under the accession number DRA002238.

### Conflict of interest statement

The authors declare that the research was conducted in the absence of any commercial or financial relationships that could be construed as a potential conflict of interest.

## References

[B1] Bartram A. K. Lynch M. D. J. Stearns J. C. Moreno-Hagelsieb G. Neufeld J. D. (2011). Generation of multimillion-sequence 16S rRNA gene libraries from complex microbial communities by assembling paired-end illumina reads. Appl. Environ. Microbiol. 77, 3846–3852. 10.1128/AEM.02772-10 21460107 PMC3127616

[B2] Basu A. Mukherjee N. Roy S. Sengupta S. Banerjee S. Chakraborty M.. (2003). Ethnic India: a genomic view, with special reference to peopling and structure. Genome Res. 13, 2277–2290. 10.1101/gr.1413403 14525929 PMC403703

[B3] Borody T. J. Brandt L. J. Paramsothy S. (2014). Therapeutic faecal microbiota transplantation: current status and future developments. Curr. Opin. Gastroenterol. 30, 97–105. 10.1097/MOG.0000000000000027 24257037 PMC3868025

[B4] Boyle R. J. Robins-browne R. M. Tang M. L. K. (2006). Probiotic use in clinical practice: what are the risks? Am. J. Clin. Nutr. 1–3, 1256–1264. 16762934 10.1093/ajcn/83.6.1256

[B5] Caporaso J. G. Kuczynski J. Stombaugh J. Bittinger K. Bushman F. D. Costello E. K. (2010). QIIME allows analysis of high- throughput community sequencing data intensity normalization improves color calling in SOLiD sequencing. Nat. Methods 7, 335–336. 10.1038/nmeth.f.303 20383131 PMC3156573

[B6] Cho I. Blaser M. J. (2012). The human microbiome: at the interface of health and disease. Nat. Rev. Genet. 13, 260–270. 10.1038/nrg3182 22411464 PMC3418802

[B7] Chung H. Pamp S. J. Hill J. A. Surana N. K. Edelman S. M. Troy E. B.. (2012). Gut immune maturation depends on colonization with a host-specific microbiota. Cell 149, 1578–1593. 10.1016/j.cell.2012.04.037 22726443 PMC3442780

[B8] David L. A. Maurice C. F. Carmody R. N. Gootenberg D. B. Button J. E. Wolfe B. E.. (2014). Diet rapidly and reproducibly alters the human gut microbiome. Nature 505, 559–563. 10.1038/nature12820 24336217 PMC3957428

[B9] De Filippo C. Cavalieri D. Di Paola M. Ramazzotti M. Poullet J. B. Massart S.. (2010). Impact of diet in shaping gut microbiota revealed by a comparative study in children from Europe and rural Africa. Proc. Natl. Acad. Sci. U.S.A. 107, 14691–14696. 10.1073/pnas.1005963107 20679230 PMC2930426

[B10] Edgar R. C. (2010). Search and clustering orders of magnitude faster than BLAST. Bioinformatics 26, 2460–2462. 10.1093/bioinformatics/btq461 20709691

[B11] Good (1953). The population frequencies of species and the estimation of population parameters. Biometrika 40, 237–264. 10.1093/biomet/40.3-4.237

[B12] Hsiao E. Y. McBride S. W. Hsien S. Sharon G. Hyde E. R. McCue T.. (2013). Microbiota modulate behavioral and physiological abnormalities associated with neurodevelopmental disorders. Cell 155, 1451–1463. 10.1016/j.cell.2013.11.024 24315484 PMC3897394

[B13] Huse S. M. Ye Y. Zhou Y. Fodor A. A. (2012). A core human microbiome as viewed through 16S rRNA sequence clusters. PLoS ONE 7:e34242. 10.1371/journal.pone.0034242 22719824 PMC3374614

[B14] Knights D. Costello E. K. Knight R. (2011). Supervised classification of human microbiota. FEMS Microbiol. Rev. 35, 343–359. 10.1111/j.1574-6976.2010.00251.x 21039646

[B15] Lahti L. Salojärvi J. Salonen A. Scheffer M. de Vos W. M. (2014). Tipping elements in the human intestinal ecosystem. Nat. Commun. 5:4344. 10.1038/ncomms5344 25003530 PMC4102116

[B16] Langille M. G. I. Zaneveld J. Caporaso J. G. McDonald D. Knights D. Reyes J. A.. (2013). Predictive functional profiling of microbial communities using 16S rRNA marker gene sequences. Nat. Biotechnol. 31, 814–821. 10.1038/nbt.2676 23975157 PMC3819121

[B17] Lazaridis I. Patterson N. Mittnik A. Renaud G. Mallick S. Kirsanow K.. (2014). Ancient human genomes suggest three ancestral populations for present-day Europeans. Nature 513, 409–413. 10.1038/nature13673 25230663 PMC4170574

[B18] Ley R. E. Hamady M. Lozupone C. Turnbaugh P. J. Ramey R. R. Bircher J. S.. (2008). Evolution of mammals and their gut microbes. Science 320, 1647–1651. 10.1126/science.1155725 18497261 PMC2649005

[B19] Ley R. E. Peterson D. A. Gordon J. I. (2006). Ecological and evolutionary forces shaping microbial diversity in the human intestine. Cell 124, 837–848. 10.1016/j.cell.2006.02.017 16497592

[B20] Lin A. Bik E. M. Costello E. K. Dethlefsen L. Haque R. Relman D. A.. (2013). Distinct distal gut microbiome diversity and composition in healthy children from Bangladesh and the United States. PLoS ONE 8:e53838. 10.1371/journal.pone.0053838 23349750 PMC3551965

[B21] Lozupone C. A. Stombaugh J. Gonzalez A. Ackermann G. Wendel D. Vázquez-Baeza Y.. (2013). Meta-analyses of studies of the human microbiota. Genome Res. 23, 1704–171714. 10.1101/gr.151803.112 23861384 PMC3787266

[B22] Lozupone C. Knight R. (2005). UniFrac: a new phylogenetic method for comparing microbial communities. Appl. Environ. Microbiol. 71, 8228–8235. 10.1128/AEM.71.12.8228-8235.2005 16332807 PMC1317376

[B23] Lozupone C. Lladser M. E. Knights D. Stombaugh J. Knight R. (2011). UniFrac: an effective distance metric for microbial community comparison. ISME J. 5, 169–1172. 10.1038/ismej.2010.133 20827291 PMC3105689

[B24] Macaulay V. Hill C. Achilli A. Rengo C. Clarke D. Meehan W.. (2005). Single, rapid coastal settlement of Asia revealed by analysis of complete mitochondrial genomes. Science 308, 1034–1036. 10.1126/science.1109792 15890885

[B25] Marathe N. Shetty S. Lanjekar V. Ranade D. Shouche Y. (2012). Changes in human gut flora with age: an Indian familial study. BMC Microbiol. 12:222. 10.1186/1471-2180-12-222 23013146 PMC3511239

[B26] Martens E. C. Koropatkin N. M. Smith T. J. Gordon J. I. (2009). Complex glycan catabolism by the human gut microbiota: the Bacteroidetes Sus-like paradigm. J. Biol. Chem. 284, 24673–24677. 10.1074/jbc.R109.022848 19553672 PMC2757170

[B27] Maurice C. F. Haiser H. J. Turnbaugh P. J. (2013). Xenobiotics shape the physiology and gene expression of the active human gut microbiome. Cell 152, 39–50. 10.1016/j.cell.2012.10.052 23332745 PMC3552296

[B28] Mellars P. Gori K. C. Carr M. Soares P. A. Richards M. B. (2013). Genetic and archaeological perspectives on the initial modern human colonization of southern Asia. Proc. Natl. Acad. Sci. U.S.A. 110, 10699–10704. 10.1073/pnas.1306043110 23754394 PMC3696785

[B29] Muegge B. D. Kuczynski J. Knights D. Clemente J. C. González A. Fontana L.. (2011). Diet drives convergence in gut microbiome functions across mammalian phylogeny and within humans. Science 332, 970–974. 10.1126/science.1198719 21596990 PMC3303602

[B30] Mueller S. Saunier K. Hanisch C. Norin E. Alm L. Midtvedt T.. (2006). Differences in fecal microbiota in different European study populations in relation to age, gender, and country : a cross-sectional study. Appl. Environ. Microbiol. 72, 1027–1033. 10.1128/AEM.72.2.1027-1033.2006 16461645 PMC1392899

[B31] Nam Y.-D. Jung M.-J. Roh S. W. Kim M.-S. Bae J.-W. (2011). Comparative analysis of Korean human gut microbiota by barcoded pyrosequencing. PLoS ONE 6:e22109. 10.1371/journal.pone.0022109 21829445 PMC3146482

[B32] Peris-Bondia F. Latorre A. Artacho A. Moya A. D'Auria G. (2011). The active human gut microbiota differs from the total microbiota. PLoS ONE 6:e22448. 10.1371/journal.pone.0022448 21829462 PMC3145646

[B33] Prasad D. S. Kabir Z. Dash A. K. Das B. C. (2011). Abdominal obesity, an independent cardiovascular risk factor in Indian subcontinent: a clinico epidemiological evidence summary. J. Cardiovasc. Dis. Res. 2, 199–205. 10.4103/0975-3583.89803 22135477 PMC3224439

[B34] Qin J. Li R. Raes J. Arumugam M. Burgdorf K. S. Manichanh C.. (2010). A human gut microbial gene catalogue established by metagenomic sequencing. Nature 464, 59–65. 10.1038/nature08821 20203603 PMC3779803

[B35] Satish Kumar R. Kanmani P. Yuvaraj N. Paari K. A. Pattukumar V. Arul V. (2013). Traditional Indian fermented foods: a rich source of lactic acid bacteria. Int. J. Food Sci. Nutr. 64, 415–428. 10.3109/09637486.2012.746288 23181843

[B36] Schloss P. D. Westcott S. L. Ryabin T. Hall J. R. Hartmann M. Hollister E. B.. (2009). Introducing mothur: open-source, platform-independent, community-supported software for describing and comparing microbial communities. Appl. Environ. Microbiol. 75, 7537–7541. 10.1128/AEM.01541-09 19801464 PMC2786419

[B37] Schluter J. Foster K. R. (2012). The evolution of mutualism in gut microbiota via host epithelial selection. PLoS Biol. 10:e1001424. 10.1371/journal.pbio.1001424 23185130 PMC3502499

[B38] Segata N. Izard J. Waldron L. Gevers D. Miropolsky L. Garrett W. S.. (2011). Metagenomic biomarker discovery and explanation. Genome Biol. 12:R60. 10.1186/gb-2011-12-6-r60 21702898 PMC3218848

[B39] Shetty S. A. Marathe N. P. Lanjekar V. Ranade D. Shouche Y. S. (2013). Comparative genome analysis of *Megasphaera* sp. reveals niche specialization and its potential role in the human gut. PLoS ONE 8:e79353. 10.1371/journal.pone.0079353 24260205 PMC3832451

[B40] Shukla H. C. Gupta P. C. Mehta H. C. Hebert J. R. (2002). Descriptive epidemiology of body mass index of an urban adult population in western India. J. Epidemiol. Commun. Health 56, 876–880. 10.1136/jech.56.11.876 12388581 PMC1732045

[B41] Subramanian S. V. Kawachi I. Smith G. D. (2007). Income inequality and the double burden of under- and overnutrition in India. J. Epidemiol. Commun. Health 61, 802–809. 10.1136/jech.2006.053801 17699536 PMC2660005

[B42] Turnbaugh P. J. Ley R. E. Hamady M. Fraser-Liggett C. M. Knight R. Gordon J. I. (2007). The human microbiome project. Nature 449, 804–810. 10.1038/nature06244 17943116 PMC3709439

[B43] Turnbaugh P. J. Ley R. E. Mahowald M. A. Magrini V. Mardis E. R. Gordon J. I. (2006). An obesity-associated gut microbiome with increased capacity for energy harvest. Nature 444, 1027–1031. 10.1038/nature05414 17183312

[B44] Valdez Y. Brown E. M. Finlay B. B. (2014). Influence of the microbiota on vaccine effectiveness. Trends Immunol. 35, 526–537. 10.1016/j.it.2014.07.003 25113637

[B45] Vecchio M. G. Paramesh E. C. Paramesh H. Loganes C. Ballali S. Gafare C. E.. (2014). Types of food and nutrient intake in India: a literature review. Indian J. Pediatr. 81(Suppl. 1), 17–22. 10.1007/s12098-014-1465-9 24928105

[B46] Wang Q. Garrity G. M. Tiedje J. M. Cole J. R. (2007). Naive Bayesian classifier for rapid assignment of rRNA sequences into the new bacterial taxonomy. Appl. Environ. Microbiol. 73, 5261–5267. 10.1128/AEM.00062-07 17586664 PMC1950982

[B47] Xing J. Watkins W. S. Hu Y. Huff C. D. Sabo A. Muzny D. M.. (2010). Genetic diversity in India and the inference of Eurasian population expansion. Genome Biol. 11:R113. 10.1186/gb-2010-11-11-r113 21106085 PMC3156952

[B48] Xu Z. Knight R. (2014). Dietary effects on human gut microbiome diversity. Br. J. Nutr. 113, S1–S5. 10.1017/S0007114514004127 25498959 PMC4405705

[B49] Yajnik C. S. Fall C. H. D. Coyaji K. J. Hirve S. S. Rao S. Barker D. J. P.. (2003). Neonatal anthropometry: the thin-fat Indian baby. The Pune maternal nutrition study. Int. J. Obes. Relat. Metab. Disord. 27, 173–180. 10.1038/sj.ijo.802219 12586996

[B50] Yatsunenko T. Rey F. E. Manary M. J. Trehan I. Dominguez-Bello M. G. Contreras M.. (2012). Human gut microbiome viewed across age and geography. Nature 486, 222–227. 10.1038/nature11053 22699611 PMC3376388

