# Supplementary material for: Molecular Characterization and Meta-Analysis of Gut Microbial Communities Illustrate Enrichment of Prevotella and Megasphaera in Indian Subjects
Source: Front Microbiol. 2016 May 9;7:660. doi: 10.3389/fmicb.2016.00660 (PMC4860526; doi:10.3389/fmicb.2016.00660)
Supplement: Supplementary file 1 [file Table1.DOCX]

**Supplementary_Table 1:** Primers used and target organisms analyzed in qPCR assays

| **Primer used** | **Sequence (5’-3’)** | **Target organism** |
| --- | --- | --- |
| 341F  518R | CCTACGGGAGGCAGCAG  ATTACCGCGGCTGCTGG | Total Bacteria |
| PrevF  PrevR | CACCAAGGCGACGATCA  GGATAACGCCYGGACCT | Prevotella |
| MegaF  MegaR | CTAGTGGCAAACGGGTGAGT  CAGACCGGCTACTGATCGTC | Megasphaera |
| FPF  FPR | GGAGGAAGAAGGTCTTGCG  AATTCCGCCTACCTCTGCACT | Faecalibacterium |
