# Supplementary material for: Molecular Characterization and Meta-Analysis of Gut Microbial Communities Illustrate Enrichment of Prevotella and Megasphaera in Indian Subjects
Source: Front Microbiol. 2016 May 9;7:660. doi: 10.3389/fmicb.2016.00660 (PMC4860526; doi:10.3389/fmicb.2016.00660)
Supplement: Supplementary file 2 [file Table2.DOCX]

**Supplementary_Table 2:** List of Primers, 16S rRNA region and sequencing technology utilized for amplicon sequencing in various studies.

| **Sr. No** | **Samples** | **Primers Used** | **Region amplified** | **Sequencing Technique** | **Reference** |
| --- | --- | --- | --- | --- | --- |
| 1. | Korean | 9F: GAGTTTGATCMTGGCTCAG  541R: WTTACCGCGGCTGCTGG | V1-V3 | Pyrosequencing | (Nam et al., 2011) |
| 2. | Bangladeshi | 27F: CATCCCTGCGTGTCTCCGACTCAG  515R: TACCGCGGCKGCTGGCAC | V1-V3 | Pyrosequencing | (Lin et al., 2013) |
| 3. | American  &  Primate | 8F: AGAGTTTGATCCTGGCTCAG  338R: TGCTGCCTCCCGTAGGAGT | V2 | Pyrosequencing | (Muegge et al., 2011) |
| 4. | Spanish | 8F: AGAGTTTGATCMTGGCTCAG  530R: CCGCGGCKGCTGGCAC | V1-V3 | Pyrosequencing | (Peris-Bondia et al., 2011) |

**References:**

Nam, Y.-D., Jung, M.-J., Roh, S. W., Kim, M.-S., and Bae, J.-W. (2011). Comparative analysis of Korean human gut microbiota by barcoded pyrosequencing. PLoS One 6, e22109. doi:10.1371/journal.pone.0022109.

Lin, A., Bik, E. M., Costello, E. K., Dethlefsen, L., Haque, R., Relman, D. a, et al. (2013). Distinct distal gut microbiome diversity and composition in healthy children from Bangladesh and the United States. PLoS One 8, e53838. doi:10.1371/journal.pone.0053838.

Muegge, B. D., Kuczynski, J., Knights, D., Clemente, J. C., González, A., Fontana, L., et al. (2011). Diet drives convergence in gut microbiome functions across mammalian phylogeny and within humans. Science 332, 970–4. doi:10.1126/science.1198719.

Peris-Bondia, F., Latorre, A., Artacho, A., Moya, A., and D’Auria, G. (2011). The active human gut microbiota differs from the total microbiota. PLoS One 6, e22448. doi:10.1371/journal.pone.0022448.
